# Spatial Organization of Acupoint Selection Across Pain Conditions

**DOI:** 10.1155/prm/2707225

**Published:** 2026-09-23

**Authors:** Heeyoung Moon, Da-Eun Yoon, In-Seon Lee, Younbyoung Chae

**Affiliations:** ^1^ Department of Meridian and Acupoints, College of Korean Medicine, Semyung University, Jecheon, South Korea, semyung.ac.kr; ^2^ Department of Meridian and Acupoints, College of Korean Medicine, Kyung Hee University, Seoul, South Korea, khu.ac.kr

**Keywords:** acupoint selection, acupuncture, distal acupoints, pain conditions, spatial organization

## Abstract

Acupuncture involves selecting acupoints distributed across the body, yet the spatial organization of this process remains unclear. Previous studies have identified frequently used acupoints across conditions but have paid limited attention to their spatial organization. Here, we analyzed patterns of acupoint selection across seven pain conditions, including episodic migraine, low back pain, dysmenorrhea, irritable bowel syndrome, carpal tunnel syndrome, osteoarthritis, and ankle sprain, using a dataset derived from randomized controlled trials. Acupoints were classified into six anatomical regions, and regional usage patterns were quantified and visualized. Acupoint selection showed two recurrent tendencies. Acupoints near the disease site were preferentially selected, reflecting region‐specific recruitment. In contrast, distal extremity points were repeatedly used across conditions, with the lower‐limb tendency being most evident in head/trunk conditions, reflecting cross‐regional recruitment. Across the seven included conditions, distal extremity regions emerged as recurrent cross‐regional components of acupoint selection. This region‐level perspective complements point‐frequency analyses by showing how acupoint selection is organized across the body.

## 1. Introduction

Acupuncture treatment relies on the selection of acupoints distributed across the body [1, 2]. In clinical practice, acupuncture prescriptions commonly combine local and distal acupoints [3]. This pattern suggests that acupoint selection is spatially organized across anatomical regions. Acupoint combinations may be associated with variations in clinical effects [4, 5]. Understanding their spatial organization may inform treatment strategies and clarify how clinical point selection relates to the underlying therapeutic mechanisms [6, 7]. Despite its clinical importance, the broader organization of acupoint selection remains insufficiently characterized.

Previous studies have applied data‐driven approaches to identify patterns of acupoint selection [8, 9]. Analyses of randomized controlled trials and acupoint networks have identified frequently used points and their co‐occurrence patterns [10, 11]. These studies reveal recurrent acupoint use but do not clarify how it is spatially related to disease location. Because point‐level analyses focus on individual acupoints, they may not fully capture broader spatial relationships between acupoint selection and disease location. A region‐level perspective is, therefore, needed to characterize acupoint selection across the body.

Previous analyses of acupuncture practices suggest that acupoint selection follows structured organizational principles, including preferential use of Five‐Shu (transport) points located in the extremities [9, 12, 13]. Classical acupuncture theory has similarly attributed broad regulatory significance to distal points, which are often used for conditions arising in distant body regions [9, 14]. Contemporary data‐driven studies also identify points such as SP6, ST36, LI4, and LR3 as frequently used across pain conditions [11, 15]. Disease‐specific network analyses have also revealed multiregional configurations combining local and distal acupoints. For example, chronic pelvic pain prescriptions integrate abdominal, lumbosacral, and distal limb points in a common network [8]. Together, these findings raise the question of whether acupoint selection follows a broader spatial organization across anatomical regions and pain conditions.

We classified acupoints into anatomical regions and quantified their regional usage patterns. We then examined whether acupoint selection was organized across the body and whether distal extremity regions were consistently recruited across conditions. We defined this dual pattern as region‐specific and cross‐regional recruitment. Region‐specific recruitment refers to greater acupoint use near the disease site, whereas cross‐regional recruitment refers to recurrent distal extremity use across conditions. This approach characterizes the spatial organization of acupoint selection across body regions at the whole‐body level.

## 2. Methods

### 2.1. Data Collection

Data on acupoint use were obtained from a previously published analysis of randomized controlled trials of acupuncture for seven pain conditions [11]. The source study extracted acupuncture treatment regimens from randomized controlled trials included in eligible Cochrane systematic reviews. Only trials with identifiable acupoint‐selection information contributed to the point‐frequency dataset.

The present study represents a secondary spatial analysis of these data. The dataset included episodic migraine (*n* = 11 trials), low back pain (*n* = 25), dysmenorrhea (*n* = 29), irritable bowel syndrome (*n* = 15), carpal tunnel syndrome (*n* = 12), osteoarthritis (*n* = 14), and ankle sprain (*n* = 7). These conditions were drawn from the source dataset and were not selected as a representative sample of all pain disorders.

For each condition, acupoint usage was expressed as the percentage of contributing trials in which each acupoint was reported. Acupoint names and locations followed the WHO standard terminology. The complete search strategy, eligibility criteria, and data extraction procedures are reported in the original publication [11].

### 2.2. Spatial Classification

Acupoints were classified into six anatomical regions: head/neck, trunk, proximal upper extremity, proximal lower extremity, distal upper extremity, and distal lower extremity. The six‐region scheme was operationally defined for the present region‐level analysis. The head/neck included cranial, facial, and cervical regions, whereas the trunk region included the chest, abdomen, back, and pelvic regions. Acupoints were further classified along a proximal–distal axis. Acupoints on the upper arm and thigh were categorized as proximal upper‐ and lower‐extremity points, respectively. Acupoints on the forearm and hand were categorized as distal upper‐extremity points, and those on the lower leg and foot were categorized as distal lower‐extremity points.

### 2.3. Quantification and Visualization

For each condition, regional usage was calculated as the mean of the point‐level usage percentage within each anatomical region. Mean values rather than summed values were used to reduce bias arising from unequal numbers of standardized acupoints across regions. Table 1 reports the resulting regional mean values and the within‐condition rank of the distal lower‐extremity region.

**TABLE 1:** Regional mean acupoint usage across seven pain conditions.

Pain condition, *n*	Head/neck	Trunk	Proximal upper	Proximal lower	Distal upper	Distal lower	Distal lower rank
Episodic migraine, *n* = 11	3.69	0.88	0	1.14	2.48	**5.4**	1
Low back pain, *n* = 25	0.7	2.04	0	**3.25**	0.58	2.94	2
Dysmenorrhea, *n* = 29	0.09	1.74	0	1.51	0.82	**4.26**	1
Irritable bowel syndrome, *n* = 15	0.36	2.39	0	0.42	2.18	**5.31**	1
Carpal tunnel syndrome, *n* = 12	0	0	1.19	0	**9.7**	0.39	3
Osteoarthritis, *n* = 14	0.1	0.15	0	**9.82**	0.78	8.15	2
Ankle sprain, *n* = 7	0.1	0	0	0	0	**9.49**	1

*Note:* Values represent the mean of the point‐level usage percentages within each anatomical region. Point‐level usage was defined as the percentage of contributing randomized controlled trials in which each acupoint was reported. Rank was calculated descriptively among the six anatomical regions within each condition. Bold values indicate the highest‐ranked region for each condition.

Spatial patterns were visualized using regional heatmaps and body‐template representations. In both visualizations, color intensity represented the mean usage percentage within each region. To examine broader spatial tendencies, conditions were additionally grouped according to their primary disease location. Episodic migraine, low back pain, dysmenorrhea, and irritable bowel syndrome were classified as head/trunk conditions. Low back pain was assigned to this group based on its primary location in the lower back region. Carpal tunnel syndrome, osteoarthritis, and ankle sprain were classified as limb conditions. Regional usage values were then averaged within each group. This operational grouping was used only for descriptive visualization and was not intended to imply that symptoms are restricted to a single anatomical region. The analysis was exploratory and descriptive, and no inferential statistical testing was performed. All data processing, regional aggregation, and visualization were performed in Python (Version 3.10).

## 3. Results

### 3.1. Spatial Organization of Acupoint Selection Across Body Regions

**FIGURE 1:**
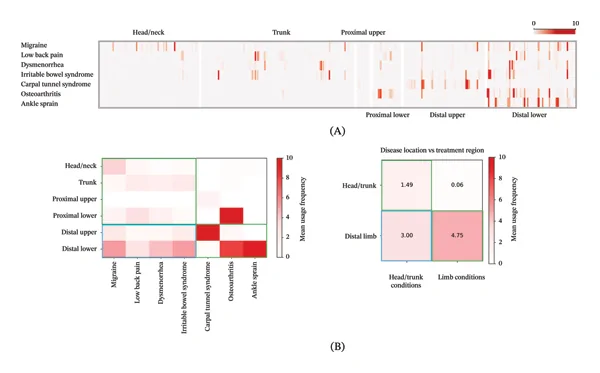
Spatial organization of acupoint selection across pain conditions. (A) Acupoint‐level heatmap showing the distribution of acupoint usage across episodic migraine (*n* = 11), low back pain (*n* = 25), dysmenorrhea (*n* = 29), irritable bowel syndrome (*n* = 15), carpal tunnel syndrome (*n* = 12), osteoarthritis (*n* = 14), and ankle sprain (*n* = 7). Acupoint usage represents the percentage of contributing randomized controlled trials in which each acupoint was reported. Acupoints are arranged according to six anatomical regions. (B) Regional heatmap summarizing the mean of the point‐level usage percentages within each anatomical region. Warmer colors indicate higher usage.

Acupoint usage across the seven pain conditions revealed a structured spatial pattern across body regions. Acupoints near the disease site were frequently used. For example, acupoints in head/neck regions were preferentially recruited in episodic migraine, whereas acupoints in trunk and lower‐extremity regions were more frequently used in low back pain and dysmenorrhea. Limb conditions showed more localized patterns. In carpal tunnel syndrome, proximal and distal upper‐extremity regions were predominantly recruited. In osteoarthritis, both proximal and distal lower‐extremity regions were prominently recruited, whereas ankle sprain predominantly involved the distal lower‐extremity region. Distal extremity acupoints were also used in conditions affecting both head/trunk and limb regions (Figure 1).

The quantitative analysis supported this regional pattern. The distal lower‐extremity region ranked first in episodic migraine, dysmenorrhea, irritable bowel syndrome, and ankle sprain; second in low back pain and osteoarthritis; and third in carpal tunnel syndrome (Table 1). In contrast, head/neck and trunk recruitment varied more clearly according to disease location.

### 3.2. Cross‐Regional Recruitment of Distal Extremity Points

**FIGURE 2:**
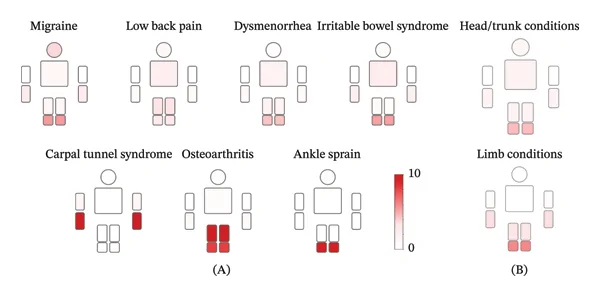
Body‐template visualization reveals cross‐regional recruitment of distal limb points. (A) Spatial distribution of mean acupoint usage across six anatomical regions for each pain condition, visualized on body templates. Warmer colors indicate higher usage. (B) Averaged spatial patterns for head/trunk conditions (episodic migraine, low back pain, dysmenorrhea, and irritable bowel syndrome) and limb conditions (carpal tunnel syndrome, osteoarthritis, and ankle sprain). Distal lower‐extremity use was particularly prominent in head/trunk conditions, illustrating cross‐regional recruitment of distal points.

Body‐template maps further illustrated how acupoint use varied with disease location. Head/trunk conditions involved acupoints from both the affected region and the extremities. Limb conditions showed more localized patterns, with predominant use of distal points and more limited use of proximal points. Distal lower‐limb points, therefore, contributed to region‐specific selection in limb conditions and cross‐regional selection in head/trunk conditions (Figure 2). Overall, acupoint selection reflected both disease‐related regional targeting and recurrent distal recruitment beyond the affected region.

## 4. Discussion

The findings reveal two complementary features of acupoint selection. Use of points near the affected region varied with condition location, whereas distal lower‐extremity points were recurrently selected across anatomically distinct conditions. This combination suggests that treatment is organized at both disease‐related and whole‐body levels. This perspective extends point‐level findings by identifying broader regional patterns of acupoint selection.

Previous disease‐specific network analyses have demonstrated multiregional configurations combining local and distal acupoints. For example, chronic pelvic pain prescriptions integrate abdominal, lumbosacral, and distal limb points in a common network [8]. The present findings extend these observations by revealing a directional asymmetry across conditions. Local points were used for both head/trunk and limb conditions, whereas cross‐regional use of distal points was most evident for head/trunk conditions. In the present dataset, remote point selection showed a predominantly centripetal pattern, with distal extremity points preferentially recruited for head/trunk conditions. The opposite tendency was less evident. The predominance of distal‐point selection for head/trunk conditions may also provide a spatial basis for the clinical importance of the Five‐Shu points. These points are located below the elbows and knees and have traditionally been used to regulate conditions in more proximal or distant regions [16]. Their distribution is, therefore, consistent with the observed recruitment of distal extremity points for head/trunk conditions. Although Five‐Shu points were not analyzed separately, this pattern may help explain their prominent role in acupuncture prescriptions.

Previous studies have identified commonly used acupoints and co‐occurrence networks across conditions [17]. Analyses of different acupuncture practices have also suggested that point selection follows structured organizational principles, including preferential recruitment of limb‐based Five‐Shu points [9]. However, these studies primarily focused on individual acupoints or pairwise relationships among points. These results suggest that repeated point use may reflect higher‐order organization across body regions. This approach complements point‐frequency and network analyses by revealing the whole‐body spatial arrangement of selected acupoints. Meridian‐specific selection represents one level of organization underlying the observed spatial patterns. Previous network analysis of acupuncture for low back pain showed predominant involvement of the Bladder Meridian. Frequent combinations included local lumbar points with distal lower‐extremity points such as BL40 and BL60 [14]. Network analysis also identified several major distal points commonly used across pain conditions [15]. Whereas these previous studies examined which acupoints and meridians were preferentially involved, the present study investigates how such selections are organized spatially across the whole body. The present findings, therefore, provide a macroscopic view that complements point‐ and meridian‐specific explanations.

The consistent use of distal limb points in anatomically distinct conditions suggests a role beyond local targeting. Clinical studies comparing distal and proximal acupoints support the functional relevance of distal stimulation, with distal acupoints providiong greater pain relief compared with proximal acupoints in knee osteoarthritis [18]. Neurophysiological studies suggest that distal limb stimulation may engage distributed pain‐modulatory systems, including descending inhibitory pathways [7, 19]. Although these mechanisms were not tested here, they provide a plausible biological context for the observed pattern. For pain research, this spatial organization highlights that treatment targets need not be confined to the anatomical site of pain. The coexistence of regional and distal point use suggests that acupuncture may engage both local pain processes and broader regulatory mechanisms. This regional perspective may inform future studies of how stimulation location relates to analgesic effects across different pain conditions.

The spatial organization identified in this study also resonates with classical theoretical frameworks in traditional East Asian medicine. Classical concepts such as root–branch (*ben–biao*) and root–knot (*gen–jie*) describe relationships between distal and proximal body regions in meridian systems [16]. In clinical practice, specific distal points are further selected according to pattern identification [5, 20]. This process integrates the patient’s signs and symptoms with relevant meridian or visceral relationships. Accordingly, distal point selection may vary among patients with the same pain condition, reflecting both disease location and the identified clinical pattern. Although not directly tested, these constructs help contextualize distal point use and the observed local–distal asymmetry. By demonstrating both disease‐related regional use and recurrent distal recruitment, this study provides insight into how local and remote stimulation are integrated in acupuncture treatment. It may also inform future empirical studies of meridian relationships, Five‐Shu points, and pattern‐based acupoint selection.

This study has several limitations. First, the analysis examined reported acupoint use rather than treatment efficacy. Second, the region‐level approach aggregates acupoints with different traditional indications and clinical roles. The prominence of a region, therefore, does not imply that all acupoints in that region are equivalent or interchangeable. The observed regional distribution may be partly driven by recurrent use of a subset of commonly selected distal points such as ST36, SP6, LR3, and LI4, which have been identified across pain conditions. Different conditions may nevertheless recruit different subsets of points. Point‐ and meridian‐level analyses should, therefore, be interpreted alongside the present regional findings. Third, classifying acupoints into six discrete anatomical regions may oversimplify their distribution. This classification scheme enabled broad spatial comparisons and descriptive visualization. However, it may not fully capture conditions with symptoms spanning multiple body areas. Fourth, the seven pain conditions were drawn from the source dataset and may not represent all pain disorders. The findings may, therefore, not generalize to pain conditions with different anatomical distributions or treatment conventions. Finally, because the dataset was derived from published randomized controlled trials, the observed patterns may partly reflect study‐design and reporting conventions and may not fully represent routine clinical practice. Future studies should integrate spatial patterns with clinical outcomes, meridian relationships, and neurophysiological measures.

## 5. Conclusion

Across the seven pain conditions, acupoint selection showed consistent disease‐related regional use together with recurrent recruitment of distal extremity points. This pattern was particularly evident in the use of distal points for head/trunk conditions and supports a whole‐body view of acupoint selection. These findings may advance understanding of traditional point‐selection principles and the spatial logic of acupuncture treatment.

## Author Contributions

Heeyoung Moon and Younbyoung Chae conceived the study; Da‐Eun Yoon, In‐Seon Lee, and Younbyoung Chae drafted the original manuscript. All authors reviewed and approved the final manuscript.

## Funding

This work was supported by the National Research Foundation of Korea (NRF) grant funded by the Korea government (MSIT) (RS‐2024‐00449485) and Korea Institute of Oriental Medicine (KSN2511011).

## Disclosure

All authors have read and agreed to the published version of the manuscript.

## Ethics Statement

The authors have nothing to report.

## Conflicts of Interest

The authors declare no conflicts of interest.

## Data Availability

The point‐level dataset, including anatomical region assignments and regional summary values, is available from the corresponding author upon reasonable request.
